# An Analysis of Lower Limb Coordination Variability in Unilateral Tasks in Healthy Adults: A Possible Prognostic Tool

**DOI:** 10.3389/fbioe.2022.885329

**Published:** 2022-06-17

**Authors:** Maryam Ghahramani, Billy Mason, Patrick Pearsall, Wayne Spratford

**Affiliations:** ^1^ Human-Centred Technology Research Centre, Faculty of Science and Technology University of Canberra, Canberra, NSW, Australia; ^2^ Faculty of Health, University of Canberra, Canberra, NSW, Australia; ^3^ University of Canberra Research Institute for Sport and Exercise Science, Canberra, NSW, Australia; ^4^ School of Information Technology and Systems, Faculty of Science and Technology University of Canberra, Canberra, NSW, Australia

**Keywords:** interlimb coordination variability, interlimb coordination, unilateral sit to stand, unilateral functional tasks, continuous hops

## Abstract

Interlimb coordination variability analysis can shed light into the dynamics of higher order coordination and motor control. However, it is not clear how the interlimb coordination of people with no known injuries change in similar activities with increasing difficulty. This study aimed to ascertain if the interlimb coordination variability range and patterns of healthy participants change in different unilateral functional tasks with increasing complexity and whether leg dominance affects the interlimb coordination variability. In this cross-sectional study fourteen younger participants with no known injuries completed three repeated unilateral sit-to-stands (UniSTS), step-ups (SUs), and continuous-hops (Hops). Using four inertial sensors mounted on the lower legs and thighs, angular rotation of thighs and shanks were recorded. Using Hilbert transform, the phase angle of each segment and then the continuous relative phase (CRP) of the two segments were measured. The CRP is indicative of the interlimb coordination. Finally, the linear and the nonlinear shank-thigh coordination variability of each participant in each task was calculated. The results show that the linear shank-thigh coordination variability was significantly smaller in the SUs compared to both UniSTS and Hops in both legs. There were no significant differences found between the latter two tests in their linear coordination variability. However, Hops were found to have significantly larger nonlinear shank-thigh coordination variability compared to the SUs and the UniSTS. This can be due to larger vertical and horizontal forces required for the task and can reveal inadequate motor control during the movement. The combination of nonlinear and linear interlimb coordination variability can provide more insight into human movement as they measure different aspects of coordination variability. It was also seen that leg dominance does not affect the lower limb coordination variability in participants with no known injuries. The results should be tested in participants recovering from lower limb injuries.

## Introduction

The biomechanical study of human motion in the fields of sports and health science can help with many aspects such as rehabilitation, injury prevention, and sports performance monitoring and analysis (Lamb and Stöckl, 2014). Many studies have modelled human movement as a dynamical system involving coordinated moving parts (Stergiou, 2004), (Glazier and Davids, 2009). The interaction and coordination of segments cause the effective displacement of the body. It is suggested that the behaviour of a dynamical system can be described by plotting a variable versus its first derivative, known as phase portraits (Rosen, 1970). Continuous relative phase (CRP) is a measure of the phase space relation of two segments evolving throughout a movement (Lamb and Stöckl, 2014), and has been widely used for inter- and intra-limb coordination analysis (Yi et al., 2016) (Lamb and Pataky, 2018) (Chopra et al., 2017). Movement coordination is associated with intersegment dynamics and the ability to construct and maintain proper coordination between joints or segments during motions (Chiu and Chou, 2012). Interlimb coordination measured by CRP has been used for injury recovery assessment such as anterior cruciate ligament reconstructive (ACLR) surgery (Goerger et al., 2020). It has also been proved that the interlimb coordination is affected by injuries, disease, and ageing (Salehi et al., 2020) (Dewolf et al., 2019) (Gueugnon et al., 2019) (Schwarz et al., 2021).

The interlimb coordination variability occurs in repeated motions. Any type of movement variability is a common inter- and intra-individual phenomena. As a movement is repeated, a certain amount of change may be recorded between its subsequent repetitions (Preatoni et al., 2013). Movement variability gives the body the degrees of freedom required to adapt to external and internal environmental conditions and to find the most appropriate movement strategies to execute a task (Preatoni et al., 2013), (Estep et al., 2018). However, increased or decreased movement variability can be indicative of lack of proper motor control caused by injury, disease or ageing (Ghahramani et al., 2020) (Thies et al., 2009), (Rennie et al., 2018). In order to properly investigate the movement variability many studies have focused on both linear and nonlinear variability assessment measures (Estep et al., 2018) (Gibbons et al., 2020). While linear methods are easy to generate and interpret, they neglect the time-dependent changes in pattern or structure of the signal (Stergiou et al., 2006), (Stergiou and Decker, 2011). Generally, motion variability is better captured by nonlinear measures where the temporal changes in the signal pattern is recorded (Stergiou and Decker, 2011). However, nonlinear variability assessment methods such as Sample Entropy (SampEn) have some limitations. This method is dependent on the sampling frequency and higher sample frequencies will lead to smaller SampEn values (Raffalt et al., 2018). Oliveira et al. (Oliveira et al., 2019) suggest that nonlinear variability assessment methods can be strong prognostic tools when used in conjunction with linear methods.

Interlimb coordination variability has been studied in sports (Floria et al., 2019). Assessing the interlimb coordination variability in repeated motions can give an insight into the stability of the system or its resiliency to perturbation. However, there is a lack of clarity on how it should be interpreted. Some studies have suggested that the coordination variability in sports is an indicator of skill, and the adaptability to generate motor patterns signifies the capability of responding to disturbances or changes in environmental conditions (Preatoni et al., 2013) (Seifert et al., 2016) (Bernstein, 1967). Meanwhile other studies found contradicting results suggesting that high coordination variability is related to an elevated injury risk or decreased level of performance due to the changes in motor control, leading to tissue overload and increased load on the joint (Hamill et al., 2012) (Seifert et al., 2013) (Mansourizadeh et al., 2020). Nevertheless, the interlimb coordination variability analysis can shed light into the dynamics of higher order coordination (Lamb and Pataky, 2018).

While most studies have assessed the coordination variability in a single activity under various conditions (i.e., walking at different speeds (Wang W. et al., 2021)), no study has assessed the interlimb coordination variability in different lower-limb movement tasks of increasing complexity. Quantifying the adjustments in interlimb coordination and coordination variability as the complexity of the functional task changes provide insights on the level of challenge required to induce changes. Modifications in coordination could indicate the need to alter the movement pattern to accomplish the demands of the new task. Changes in the coordination variability in healthy adults might indicate the degree of adaptability that is required to respond to new constraints in the task. On the other hand, increased coordination variability can be indicative of perturbed coordination beyond stability (Kelso, 1995). Hence, it is not clear how the interlimb coordination of people with no known injuries change in similar activities with increasing difficulty. Elucidating how the coordination variability changes in response to different functional activities with similar motion may help us further understand the role of variability in human movement.

The present study focuses on assessing the interlimb coordination variability in a group of adults with no known injuries in a set of unilateral functional tasks with increasing complexity. All unilateral activities consist of knee extension-flexion. In this study the two following questions are meant to be answered: 1- Does the interlimb coordination variability range and patterns of participants with no injuries change in different unilateral functional tasks with increasing complexity? and 2- Does the leg dominance affect the shank-thigh coordination variability in participants with no known injuries? We hypothesized that the interlimb coordination variability increases in tasks with less ecological validity. We also hypothesized that the interlimb coordination variability of the nondominant leg is greater than the dominant one. In order to carefully characterize the shank-thigh coordination variability in the three functional tasks both linear and nonlinear motion variability measures were used.

Therefore, the contributions of this study are: 1) assessing the linear interlimb coordination variability of participants in the three different functional tasks, 2) assessing the nonlinear interlimb coordination variability, 3) comparing the linear and nonlinear interlimb coordination variability of the participants in the functional tasks with increasing difficulty and complexity 4) assessing the effect of leg dominance on the shank-thigh coordination variability in the three different tasks. The three functional tasks in this study have been selected as they have similar knee extension-flexion motion and due to their application in clinical settings (McQuade and De Oliveira, 2011) (Jonsson and Kärrholm, 1994), as assessments of lower-limb strength (Waldhelm et al., 2020), or due to their similarities to activities of daily living (Grimmer et al., 2020). The results of this study indicate that participants displayed different results in their linear and nonlinear coordination variability. It was also seen that despite our initial hypothesis the leg dominance does not affect the interlimb coordination variability in participants with no known injuries. It is anticipated that the findings of this study may be used as a baseline for future studies investigating interlimb coordination in participants recovering from lower limb injuries such as ACL.

## Methods

### Study Participants

Fourteen younger adult participants with no known injuries aged 18–35 (28 ± 7.69) years were recruited to participate in this study and provided informed consent prior to their participation. Upon arrival to the testing facility participants completed an Adult Pre-Exercise Screening System (APSS; Exercise and Sports Science Australia). Using the APSS tool any participant with injury, known disease or symptoms of disease that would have impacted their ability to complete the required movement tasks were identified and excluded from the study.

### Study Experiments

This study used a cross-sectional study design to assess coordination variability in three unilateral movement tasks with increasing complexity. Participants’ dominant leg was determined by asking participants which leg they would kick a soccer ball with or land from a jump (Weir et al., 2019). Upon arrival to the testing facility participants completed 5-min on a cycle ergometer to warm up the musculature of the lower-body using a previously described protocol (Kelly et al., 2010). Once the warm-up was completed, participants performed a single practice set of the unilateral sit-to-stand (UniSTS), unilateral step-ups (SUs), and three continuous-hops (Hops) before being fitted with inertial sensors (MTw from Xsens Technology). Once the sensors were applied, each participant completed the UniSTS, SUs and Hops in a randomized order using the techniques outlined below. As we were aiming at assessing the interlimb coordination variability three repetitions were included in each task. We also conducted two trials for each task to avoid any possible data loss due to technical issues.

**FIGURE 1:**
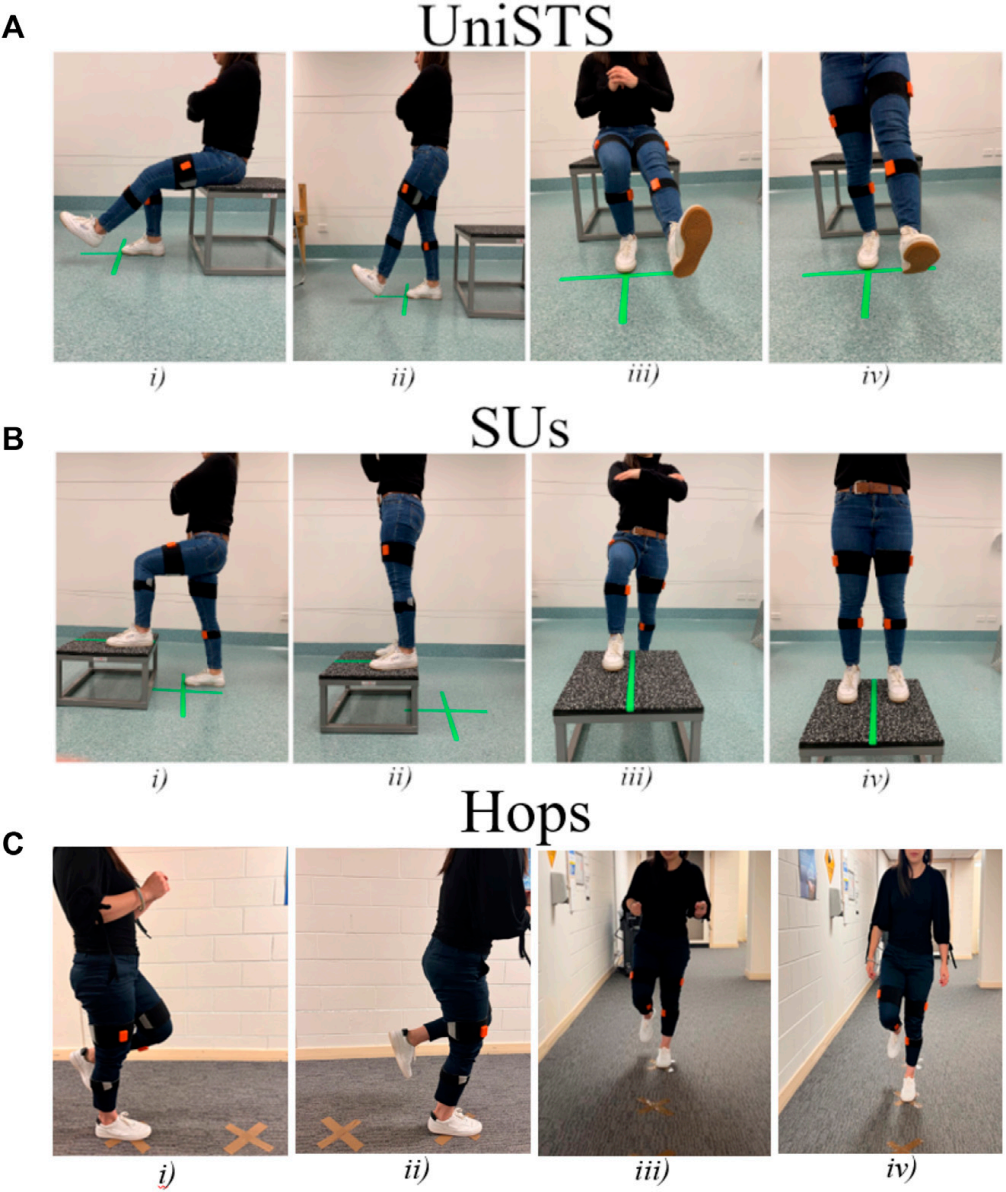
The standardization procedures for **(A)** UniSTS, **(B)** SUs, and **(C)** Hops in starting and ending position, with a sagittal plane view and frontal plane view. Two inertial sensors were attached to the shanks and two to the thighs of the participants.

In the UniSTS participants started from a seated position on a box. They placed a single foot on the ground at the midline of the body with arms across their chest. Participants were instructed to stand to an upright position before returning to the seated position on the box in a controlled manner. The UniSTS was performed for 2 sets of three repetitions each leg (Figure 1A). The SUs commenced with a leg placed on top of a 30 cm box, with the other foot placed flat on the floor behind the box as outlined in Figure 1B. The box was positioned to allow for 90-degree knee flexion in the front leg when in the start position. When instructed, participants applied pressure through their front foot to bring their rear foot onto the box and finish in a tall standing position. The rear foot was then removed from the box and returned to the floor with the participant finishing the movement back in the start position ready to perform the next repetition. This process was repeated for 2 trials and each trial consisted of a set of three repetitions each leg. The Hops were performed by landing at markers placed 50 cm apart (Figure 1C). Whilst hop tests are often performed as a performance task with the aim of covering maximal distance (Ageberg and Cronström, 2018), the distance was set at 50 cm in the current study to allow for intra- and inter-trial standardization. This approach also ensured that data observed was due to differences in interlimb coordination and not distance covered during the task. Participants were instructed to jump forwards off a single leg and land on the same leg for a total of three repetitions each side. The Hops were performed in a continuous linear motion and were repeated for a total of two sets of three continuous repetitions each side.

### Measurement System

Four inertial sensors (MTw from Xsens Technology) were attached on each participant; two on the shanks, placed on the medial surface of the tibia and two on the thighs placed on the lateral side superior to the knee joint. The shank and thigh angular rotation in the sagittal plane were recorded with the sampling rate of 60 Hz before data was transmitted to a computer for further analysis using MATLAB (R2021a). To ensure that extra movements did not affect the analysis, any data points recorded before and after the movement tasks were removed by reviewing the recorded sessions in Xsens MVN Analyze software. The data was pre-processed within MVN analyze which applies a Kilman filter to the data.

In order to assess the shank-thigh coordination variability, the phase angle of the two segments must first be determined (Ippersiel et al., 2018). In a similar method to Lamb and Stockl (Lamb and Stöckl, 2014) the amplitude of each segment angular rotation data is firstly centered around zero. Having the segment angular rotation θ(t) the centered angular rotation for the time $t_{i}$ noted as $\theta_{centred}\left(t_{i}\right)$ is as follows:

$$\theta_{centred}\left(t_{i}\right)=\theta \left(t_{i}\right)-\min\left(\theta \left(t\right)\right)-\frac{\max\left(\theta \left(t\right)\right)-\text{min}\left(\theta \left(t\right)\right)}{2}$$ (1)

min(θ(t)) and max(θ(t)) are indicative of the minimum and the maximum value of θ(t) respectively. Subsequently each centered angular rotation set is transformed into a complex analytical signal of ζ(t) using the Hilbert transform (Ippersiel et al., 2018).

$$\text{ζ}\left(\text{t}\right)={\text{θ}}_{centered}\left(\text{t}\right)+\text{ iH}\left(\text{t}\right)$$ (2)

The Hilbert transform of signal U(t) is the convolution of U(t) with the signal h $\left(t\right)=\frac{1}{\pi t}$ . The Cauchy principal value is used in the Hilbert Transform (Gabor, 1946). Using Hilbert transform, the phase difference between two arbitrary, non-stationary, non-sinusoidal signals can be determined. The phase angle at each time point $t_{i};\emptyset \left(t_{i}\right)$ is calculated as the inverse tangent of imaginary part of the complex analytical representation of the segment data ζ(t) divided by its real part.

$$\emptyset \left(t_{i}\right)=\text{arctan}\left(\frac{H\left(t_{i}\right)}{\theta_{centered}\left(t_{i}\right)}\right)$$ (3)

Continuous relative phase (CRP) analysis was then used to describe patterns of phase relationship between the two segments’ angular rotation as the difference between the phase angle of each segment at $t_{i}$ :

$$CRP\left(t_{i}\right)=\emptyset_{1}\left(t_{i}\right)-\emptyset_{2}\left(t_{i}\right)$$ (4)

A value of 0° for the CRP of the segments is indicative of them being fully in-phase, whereas CRP of 180° represents a fully out-of-phase coupling. CRP shows how the two segments are coupled in their movements during the functional task (Stergiou et al., 2001). When two segments are out-of-phase they are moving in opposite directions and two in-phase segments move in a similar fashion and are totally coordinated. As stated by Lamb and Stockl (Lamb and Stöckl, 2014), in order to find meaningful and interpretable results to describe phase relationships properly from a dynamical systems perspective only segment angles should be used for calculating the CRP. For this purpose, the CRP for the shank-thigh segments was calculated.

**FIGURE 2:**
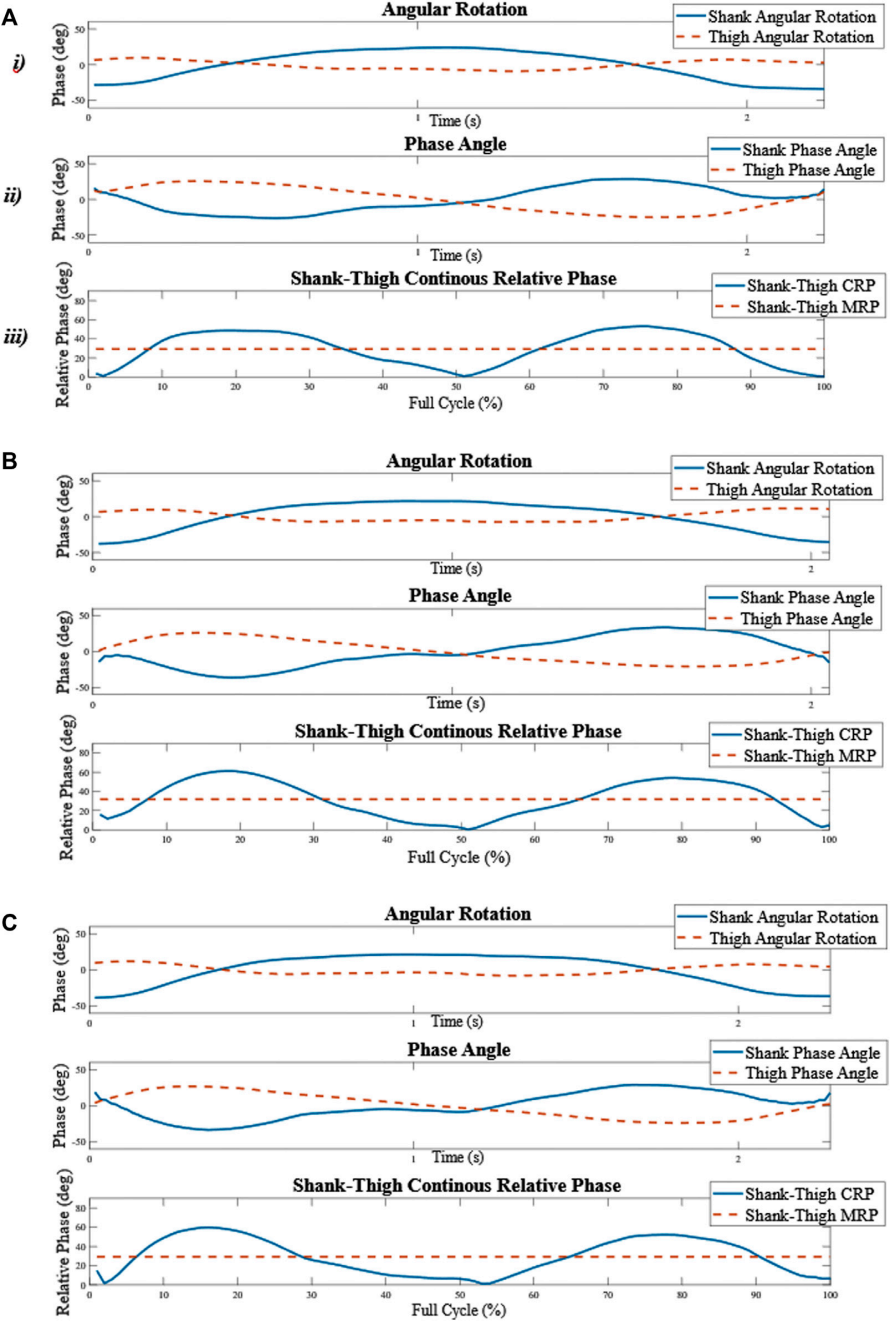
Schematic overview of the calculation of the shank-thigh coordination measures. **(A)** *i*) The angular rotation of the right thigh and lower leg in the sagittal plane in one repetition of the SUs. **(A)** *ii*) The right shank-thigh continuous relative phase (CRP) over the full cycle of one repetition of the SUs. **(A)** *iii*) The mean of the CRP over the functional cycle is calculated and averaged for each repetition in each functional task for each participant and is referred to as the MRP. **(B–C)** The process was repeated for the second and the third repetitions. The standard deviation of the MRP over the three repetitions in every task is referred to as the linear coordination variability (sdMRP).

The mean CRP over each repetition in every functional task is referred to as the mean relative phase (MRP). Finally, the standard deviation of the MRP (sdMRP) over the different repetitions in every functional task was calculated to assess linear coordination variability (Meyns et al., 2020). A schematic overview of the calculation of shank-thigh coordination measures is shown in Figure 2.

Sample Entropy (SampEn) is chosen for the nonlinear coordination variability analysis (Delgado-Bonal and Marshak, 2019). SampEn looks into the probability of two or more adjacent values in a time series being used to predict the next value. If SampEn is applied to a periodic signal the result will be 0, and if SampEn is above 1 it is indicative that the signal is with little to no clear periodic patterns. In order to calculate nonlinear coordination variability, firstly the shank-thigh CRP in each repetition in each functional task (CRP_1_-CRP_3_) was arranged in a vector as:

X=[CRP1;CRP2;CRP3] (5)

Then SampEn was applied to X. SampEn can be defined as:

$$SampEn\left(m,r,N\right)=-\ln\left(\frac{A}{B}\right)$$ (6)

**FIGURE 3:**
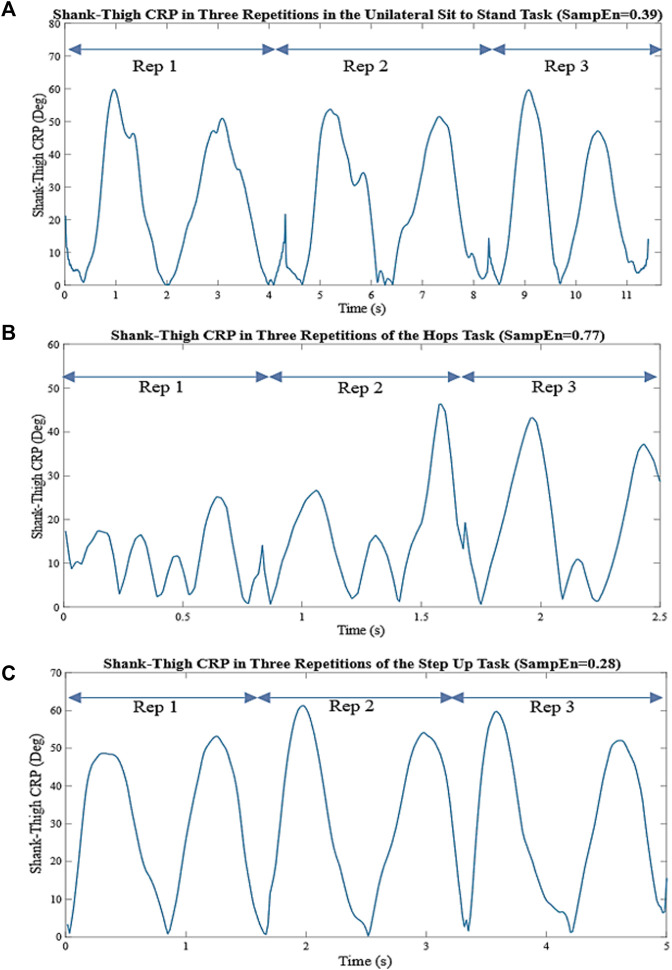
**(A)** Shank-thigh CRP in three repetitions of the UniSTS. **(B)** Shank-thigh CRP in three repetitions of the Hops. **(C)** Shank-thigh CRP in three repetitions of the SUs. The nonlinear coordination variability is measured by the SampEn applied to the overall shank-thigh CRP in all repetitions. The higher SampEn is indicative of larger interlimb coordination variability and irregularity.

Where m is the embedded dimension (typically 2 for most cases); r is the tolerance interval (typically the standard deviation of the signal multiplied by 0.2); and N is the length of the signal X. A is defined as the number of vector pairs having $d\left[X_{m+1}\left(i\right),\;\;\;X_{m+1}\left(j\right)\right]<r$ and B as the number of vector pairs having $d\left[X_{m}\left(i\right),\;\;\;X_{m}\left(j\right)\right]<r$ . Xm(i) and Xm(j) are vector pairs of length m where j ≠ i to avoid self-counting, and the d [·] denotes the distance function (Delgado-Bonal and Marshak, 2019). In Figure 3. the shank-thigh CRP of a participant in three repetitions of the UniSTS, Hops, and SUs and their relative SampEn values are shown.

### Statistical Analysis

Using the Kolmogorov-Smirnov test the normality assumption of the results was tested. As most results rejected the null hypothesis (*p* < 0.05) non-parametric tests were used. Using the Friedman test, followed by Wilcoxon non-parametric test the significant differences in each leg’s coordination variability in different functional tests were investigated. The effect size r was calculated and considered as small for r < 0.1, medium for 0.1 < r < 0.3, and large for r > 0.5 (Rosenthal, 1994). Significant differences of the linear and nonlinear variability results of the dominant leg compared to the nondominant leg were investigated using Kruskal-Walis ANOVA. The significance level was set by a *p*-value below 0.05.

## Results

Firstly, the right shank-thigh linear coordination variability is compared in the UniSTS, Hops, and SUs test using the Friedman test. The shank-thigh linear coordination variability of the right leg is found significantly different in the UniSTS, Hops, and SUs (*χ* ^*2*^ *(2) =*7.00, *p* = 0.03). Similarly, the shank-thigh non-linear coordination variability of the right leg is found significantly different in the UniSTS compared to that in Hops, and SUs (*χ* ^*2*^ *(2)* = 23.29, *p* < 0.001). The same process was repeated for the left leg. The shank-thigh linear coordination variability of the left leg is found significantly different in the three tests (*χ* ^*2*^ *(2) =*10.71, *p* = 0.005). The left leg’s nonlinear shank-thigh coordination variability is found significantly different in the UniSTS, Hops, and SUs (*χ* ^*2*^ *(2)* = 24.15, *p* < 0.001).

**FIGURE 4:**
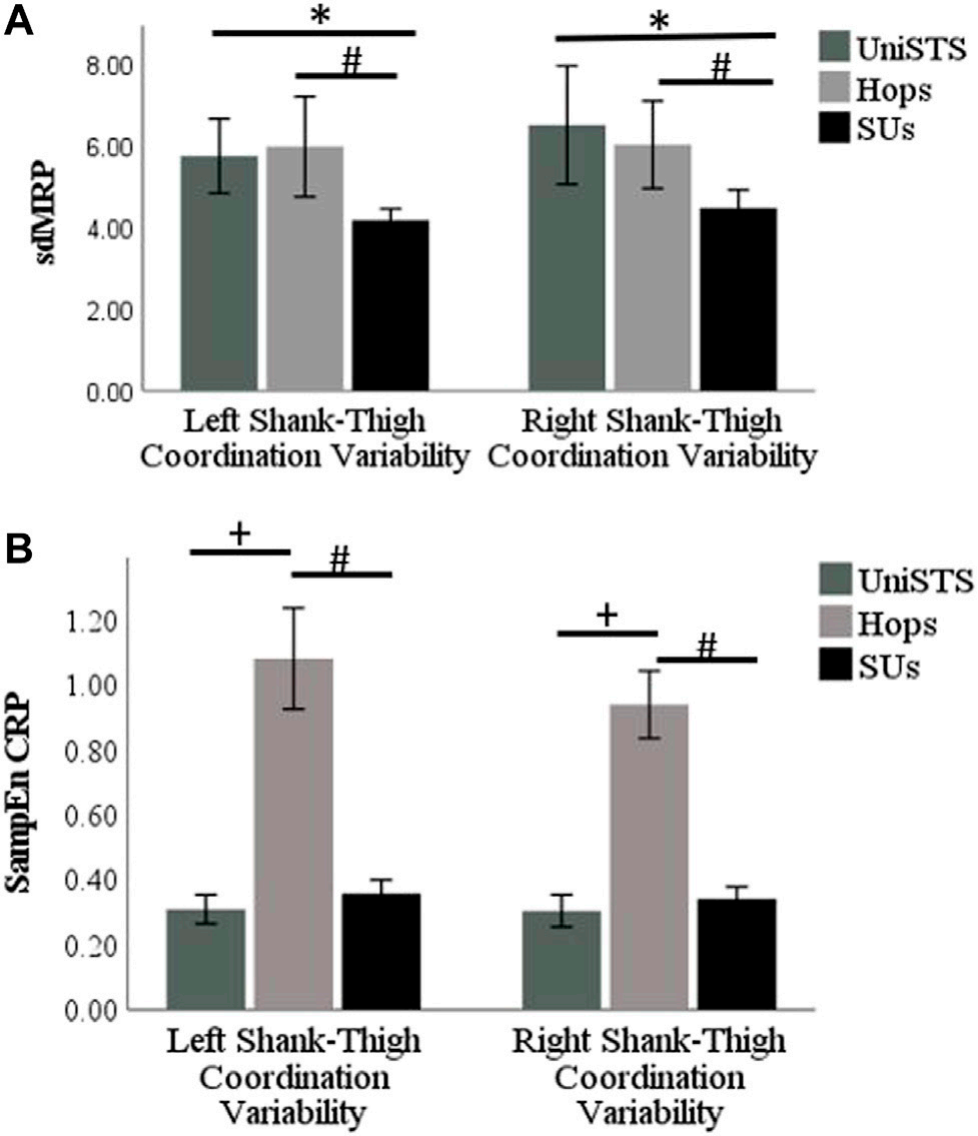
**(A)** The bar plot of the linear right and left shank-thigh coordination variability results measured by the sdMRP of the participants in UniSTS, SUs, and Hops. **(B)** The bar plot of the non-linear right and left shank-thigh coordination variability results measured by the SampEn CRP of the participants in UniSTS, SUs, and Hops. The significant differences (*p* < 0.05, *r* > 0.5) are shown by * for UniSTS vs. SUs, ^#^ for Hops vs. SUs, and ^+^ for UniSTS vs. Hops.

The median and the confidence interval of the shank-thigh coordination variability results for each leg in different tasks and the results of the Wilcoxon signed-rank test for pairwise comparison are shown in Table 1. The bar plot of the results on the linear and nonlinear right and left shank-thigh coordination variability are shown in Figure 4.

**TABLE 1:** The right and left shank-thigh linear and nonlinear coordination variability analysis results in the UniSTS, Hops, and SUs and the Wilcoxon signed-rank test results.

	UniSTS	Hops	SUs
Right leg			
*sdMRP*	5.36 (4.81–6.63)*	5.66 (4.72–7.17)^#^	4.23 (3.86–4.43)*^#^
	*p = 0.009		^*#*^ *p = 0.009*
	*Z = −*2.6*		^*#*^ *Z =* −*2.6*
	*r = 0.69		^*#*^ *r = 0.69*
*SampEn CRP*	0.29 (0.25–0.35)^**+**^	0.92 (0.83–1.04)^**+**#^	0.35 (0.30–0.37)^#^
	^**+**^ *p ≤ 0.001*	^*#*^ *p ≤ 0.001*	
	^**+**^ *Z =* −*3.3*	^*#*^ *Z =* −*3.3*	
	^**+**^ *r = 0.88*	^*#*^ *r = 0.88*	
Left leg			
*sdMRP*	5.81 (5.03–7.91)*	5.67 (4.93–7.06)^#^	4.21 (4.01–4.89)*^#^
	*p = 0.002		^*#*^ *p* = 0.009
	*Z = −3.2		^*#*^Z = −2.6
	*r = 0.85		^*#*^r = 0.69
*SampEn CRP*	0.29 (0.26–0.35)^**+**^	0.98 (0.92–1.23)^**+**#^	0.33 (0.31–0.4)^#^
	^**+**^ *p* ≤ 0.001	^#^ *p* ≤ 0.001	
	^**+**^ *Z* = −3.3	^*#*^ *Z* = −3.2	
	^**+**^ *r* = 0.88	^*#*^ *r* = 0.85	

CI, confidence interval; sdMRP, standard deviation of the mean relative phase; SampEn CRP, sample entropy of the continuous relative phase. The significant differences (*p < 0.05*, *r > 0.5*) are shown by * for UniSTS vs. SUs, ^#^ for Hops vs. SUs, and ^+^for UniSTS vs. Hops.

In another comparison in order to test the effect of leg dominance on the coordination variability the dominant and nondominant shank-thigh coordination variability were compared separately within each individual functional task. Despite our initial hypothesis, the effect of the leg dominance on the shank-thigh coordination variability is not apparent. No significant differences were found in the linear shank-thigh coordination variability of the dominant leg compared to the nondominant one in any functional task. Similarly, the nonlinear shank-thigh coordination variability did not show any significant differences when compared in the dominant leg to the non-dominant one in any of the tasks (Table 2).

**TABLE 2:** Linear and nonlinear coordination variability analysis of the dominant and nondominant legs results in the UniSTS, Hops, and SUs.

	Dominant leg	Non-Dominant leg
*sdMRP Median (CI)*		
UniSTS	5.6 (5.03–7.56)	5.35 (4.73–7.03)
Hops	5.98 (5.08–7.5)	5.52 (4.6–6.7)
SUs	4.26 (3.9–4.46)	4.12 (3.96–4.87)
*SampEn CRP Median (CI)*		
UniSTS	0.29 (0.26–0.35)	0.28 (0.25–0.35)
Hops	0.98 (0.93–1.25)	0.90 (0.84–1.00)
SUs	0.33 (0.31–0.38)	0.37 (0.31–0.39)

CI, confidence interval; sdMRP, standard deviation of the mean relative phase; SampEn CRP, sample entropy of the continuous relative phase.

## Discussion

This study focused on the shank-thigh coordination variability in different unilateral functional tasks in adults with no known injuries. In this study both the linear and nonlinear shank-thigh coordination variability were assessed. The results of shank-thigh sdMRP is significantly smaller in the SUs compared to both UniSTS and the Hops in both legs. However, the results yielded from both right and left shank-thigh SampEn are significantly larger in the Hops compared to both UniSTS and SUs. This difference in the nonlinear and the linear coordination variability shows that they cover different aspects of human motion. In order to have better understanding on the interlimb coordination variability and its role in human movement, it is beneficial to assess both measures. It was also found that the dominant shank-thigh coordination variability did not have any significant differences compared to the nondominant shank-thigh coordination variability in any of the tasks. The results of this study can be used as a baseline for future studies investigating interlimb coordination in participants recovering from lower limb injuries. A better understanding of the range of coordination variability in healthy adults aids with the injury recovery monitoring and physiotherapy.

Motor control associates with intersegmental dynamics and the ability to construct and maintain proper coordination between joints or segments during motions (Chiu and Chou, 2012) (Needham et al., 2014). Analyzing the interlimb coordination variability can help with understanding the dynamics of higher order coordination and to investigate the stability of the human movement system, or its resiliency to perturbation. (Lamb and Stöckl, 2014). Most studies focus on one activity with different speed or under different condition (Wang Y. et al., 2021). To the best of our knowledge this is the first study to examine shank-thigh coordination variability in unilateral tasks with knee extension-flexion of increasing complexity. According to Clark et al. (Clark et al., 1993) shank-thigh coordination is cyclic and dissipative and therefore energy must be supplied to continue the behavior. The three functional tasks with similar knee extension-flexion have different difficulty levels that can act as external constraints. Any external constraint can cause the fluctuation in the stability of human motion system and affect the interlimb coordination (Glazier et al., 2006).

The linear shank-thigh coordination variability in the SUs is found to be significantly smaller compared to UniSTS and the SUs in both right and left legs. This could be due to the level of familiarity the participants have with the SUs movement compared to the UniSTS and the Hops. The refined motor control process required for consistent movement is developed through a learning process (Guarrera-Bowlby and Gentile, 2004). As the motor control adopts a functionally preferred state of interlimb coordination, the dynamics of order parameters are highly ordered and stable leading to the consistent patterns of interlimb coordination (Kelso, 1995). The step-up is a closed chain movement which mimics stair ascent and other common activities of daily living (Pijnappels et al., 2010). However, both dynamic hops and the open chain UniSTS tasks are of less ecological validity.

In addition to the linear interlimb coordination variability analysis, we assessed the nonlinear coordination variability using SampEn. While most studies rely on linear interlimb coordination variability analysis, non-linear measures of variability are found to be a potentially powerful prognostic tool when used in conjunction with linear measures (Oliveira et al., 2019). In the nonlinear shank-thigh coordination variability, Hops are found to have significantly larger SampEn results, i.e., larger nonlinear shank-thigh coordination variability compared to the SUs and the UniSTS. There are some everyday situations, e.g., car egress, which are to some extent similar to the unilateral sit-to-stand (Steingrebe et al., 2018). However, continuous unilateral hopping is far less common in activities of daily living. Moreover, during the Hops larger forces and acceleration in the vertical and anterior-posterior directions are needed compared to SUs and UniSTS to move the body and the center of mass both horizontally and vertically. The nonlinear interlimb coordination variability assessed by the SampEn investigates the irregularity of the patterns of the signal through repetitions. When it comes to the interlimb coordination, the ability to have more regular patterns is more difficult in complex or less familiar tasks (as seen in Figure 4). While larger motion variability is not always indicative of lack of stability, the large irregularity of interlimb coordination patterns in the Hops in the absence of any external perturbance can be considered as a lack of proper motor control (Van Emmerik et al., 2016). This result suggests that in addition to the common linear interlimb coordination variability (sdMRP), the nonlinear assessment of the coordination variability by SampEn should be considered.

Despite our initial hypothesis our results indicate that leg dominance did not have any effect on the coordination variability in participants with no known injuries. There were no significant differences in the shank-thigh coordination variability of participants’ dominant and nondominant legs in any of the unilateral functional tasks. This result indicates symmetry in shank-thigh coordination variability of participants regardless of the task. Similar to our results, it is reported by some studies that in healthy adults, leg dominance does not influence lower limb functionality (McGrath et al., 2016), (Greska et al., 2017), (Steingrebe et al., 2018). It is anticipated that injured participants show asymmetry in their dominant and nondominant lower limb coordination variability specifically in their nonlinear coordination variability. In a pilot study by Albano et al. (Albano et al., 2021) uninjured participants showed symmetrical knee joint variability. Meanwhile, the injured participants with no rehabilitation showed a noteworthy asymmetry. This result is interesting in itself and should be tested in participants recovering from lower limb injuries.

### Limitation and Future Work

The findings of this study should be interpreted in light of some limitations. One limitation of this study is that some participants had more active lifestyles compared to others within the sample. This might have affected their range of coordination variability specially in the Hops and the UniSTS which are more demanding tasks compared to the SUs. It would be beneficial to repeat this study on an athletic population. In a study by Wang et al. (Wang W. et al., 2021) athletes showed smaller coordination variability while running compared to nonathletes suggesting that coordination variability may depend on motor skill level. Another limitation is that the height and the mass of the participants were not considered in the analysis. These factors might have affected the shank-thigh coordination variability results. In future studies other general and pathological factors of the participants that might affect their interlimb coordination should be taken into consideration.

## Conclusion

The results of this study show that the combination of nonlinear and linear interlimb coordination variability analysis can provide more information on human movement. Participants had significantly smaller linear shank-thigh coordination variability in the SUs when compared to the UniSTS and Hops. There were no significant differences found between the linear interlimb coordination variability of the latter two tasks. However, nonlinear coordination variability is found to be significantly larger in the Hops compared to the two other tasks. This indicates irregularity in the interlimb coordination patterns which can be due to the larger vertical and horizontal forces required for the task, which may in turn reveal inadequate motor control during the movement. It was also seen that leg dominance does not affect the interlimb coordination of the participants with no known injuries. The results of this study should be tested on athletes with more familiarity in unilateral functional tasks and can be used as a benchmark for participants recovering from lower limb injuries.

## Data Availability

The raw data supporting the conclusions of this article will be made available by the authors, without undue reservation.
